# Ictal Occurrence of High-Frequency Oscillations Correlates With Seizure Severity in a Rat Model of Temporal Lobe Epilepsy

**DOI:** 10.3389/fnhum.2021.624620

**Published:** 2021-06-08

**Authors:** Nadja Birk, Jan Schönberger, Karin Helene Somerlik-Fuchs, Andreas Schulze-Bonhage, Julia Jacobs

**Affiliations:** ^1^Department of Neuropediatrics and Muscle Disorders, Medical Center—University of Freiburg, Freiburg, Germany; ^2^Epilepsy Center, Medical Center—University of Freiburg, Freiburg, Germany; ^3^Berta-Ottenstein-Programme, Faculty of Medicine, University of Freiburg, Freiburg, Germany; ^4^Department of Paediatrics and Department of Neuroscience, Cumming School of Medicine, University of Calgary, Calgary, AB, Canada; ^5^Hotchkiss Brain Institute and Alberta Children’s Hospital Research Institute, University of Calgary, Calgary, AB, Canada

**Keywords:** high frequency oscillations, epileptic seizures, seizure severity, Racine scale, mesio-temporal epilepsy

## Abstract

High-frequency oscillations (HFOs, ripples 80–250 Hz, fast ripples 250–500 Hz) are biomarkers of epileptic tissue. They are most commonly observed over areas generating seizures and increase in occurrence during the ictal compared to the interictal period. It has been hypothesized that their rate correlates with the severity of epilepsy and seizure in affected individuals. In the present study, it was aimed to investigate whether the HFO count mirrors the observed behavioral seizure severity using a kainate rat model for temporal lobe epilepsy. Seizures were selected during the chronic epilepsy phase of this model and classified by behavioral severity according to the Racine scale. Seizures with Racine scale 5&6 were considered generalized and severe. HFOs were marked in 24 seizures during a preictal, ictal, and postictal EEG segment. The duration covered by the HFO during these different segments was analyzed and compared between mild and severe seizures. HFOs were significantly increased during ictal periods (*p* < 0.001) and significantly decreased during postictal periods (*p* < 0.03) compared to the ictal segment. Ictal ripples (*p* = 0.04) as well as fast ripples (*p* = 0.02) were significantly higher in severe seizures compared to mild seizures. The present study demonstrates that ictal HFO occurrence mirrors seizure severity in a chronic focal epilepsy model in rats. This is similar to recent observations in patients with refractory mesio-temporal lobe epilepsy. Moreover, postictal HFO decrease might reflect postictal inhibition of epileptic activity. Overall results provide additional evidence that HFOs can be used as biomarkers for measuring seizure severity in epilepsy.

## Introduction

High-frequency oscillations (HFOs) between 80 and 500 Hz are biomarkers for epileptic tissue. They were described recorded with intracranial microelectrodes and closely linked to mesio-temporal structures that generated spontaneous seizures (Bragin et al., 1999). Later, it could be shown that both ripples (80–250 Hz) and fast ripples (250–500 Hz) were linked to the seizure onset zone (SOZ) in patients with refractory epilepsy (for review, see Frauscher et al., 2017). Moreover, surgical removal of HFO-generating areas was associated with a higher likelihood of seizure freedom after epilepsy surgery (Jacobs et al., 2010, 2018; Wu et al., 2010). Most of the studies investigating whether HFOs could help to localize epileptic tissue focused on the analysis of interictal HFOs. However, ictal gamma oscillations and HFOs can also be identified during epileptic seizures (Jirsch et al., 2006; Jacobs et al., 2009; Weiss et al., 2015; Smith et al., 2020). It is known that HFO activity increases during seizures and that this increase is most prominent in the SOZ (Jirsch et al., 2006; Weiss et al., 2013). Studies have also tried to find systematic HFO increases prior to the seizure onset; however, results are currently conflicting (Jirsch et al., 2006; Khosravani et al., 2009; Jiruska et al., 2017), and no systematic approach for seizure prediction could be found using HFOs. It could also be shown that HFOs increase systematically at seizure onset independent of the seizure onset pattern and etiology (Perucca et al., 2014).

In the recent decade, increasing evidence was found that HFOs not only can be used to localize epileptic activity but also can directly reflect seizure propensity and disease severity in patients with epilepsy (Zijlmans et al., 2009). Thus, successful treatment with various antiepileptic drugs resulted in HFO reduction in patients as well as rodent animal models (for review, see Lévesque et al., 2018). HFO occurrence can be used as a predictor of seizure severity in several pediatric epilepsy syndromes (Kobayashi et al., 2015; van Klink et al., 2016). We could show that HFO occurrence around the seizure could mirror seizure severity in patients with mesio-temporal lobe epilepsy (Schönberger et al., 2019). However, in patients with mesio-temporal epilepsy, clinical assessment of seizure severity is challenged by the large variability of observed seizures. Seizure classifications successfully distinguish between seizure with and without secondary bilateral involvement, but no simple scale can be applied to assess seizure severity in patients.

The kainate rat model is an animal model for mesio-temporal epilepsy that allows studying chronic epilepsy with seizures of varying severity in a controlled environment (for review, see Lévesque and Avoli, 2013). The kainate injection provokes neuronal loss and cell dispersion resembling hippocampal sclerosis as seen in patients with temporal lobe epilepsy (Suzuki et al., 1995).

Observed spontaneous seizures show very similar semiology as in patients and can be classified, according to a modified Racine scale, into six different degrees of clinical seizure severity depending on semiology (Racine, 1972). We investigate HFO occurrence during the preictal, ictal, and postictal periods in rats with focal mesio-temporal epilepsy and hypothesized that seizure severity is mirrored by HFO occurrence.

## Materials and Methods

### Animal Selection and Preparation

Adult female and male Wistar rats (250–350 g, <1 year) were used for this study. All procedures described in this study were approved by the Institutional Animal Care and Use Committee of the University of Freiburg, and experiments were performed in accordance with the relevant guidelines and regulations. It is important to note that the presented dataset was used retrospectively, and animals were originally undergoing these experiments for a separate project.

### Kainate Injection and Implantation of Intracranial Electrodes

Animals were anesthetized with ketamine (100 mg/kg), xylacine (3 mg/kg), and atropine (0.1 mg/kg) and fixed into a stereotactic frame. A guide cannula was implanted into the right posterior hippocampus with the following stereotactic coordinates: AP −5.5 L −4.8 V −5.0 with regard to Bregma. Kainic acid was injected into the hippocampus by means of a Hamilton syringe, applying 0.4 μg/0.1 μl continuously over 1 min. Established status was confirmed by EEG recordings. Status epilepticus was interrupted after 2 h using diazepam.

**Figure 1:**
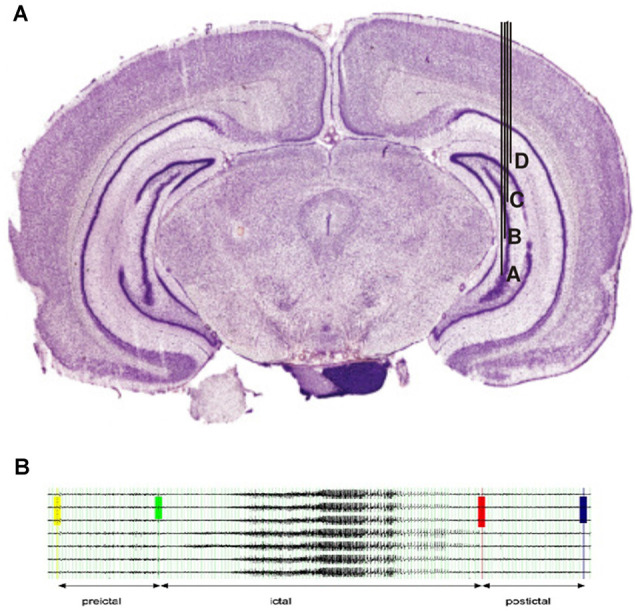
**(A)**Localization of electrodes in the right hippocampus. Injection of kainate acid was placed between contacts (B&C). **(B)** Example for the selection of EEG segments in a typical seizure.

This was followed by implanting a recording electrode with a depth of 6.5 mm, resulting in the four contacts of the electrode surrounding the region of kainate injection (see Figure 1A for electrode placement). The electrode consisted of 125-μm-thick Teflon-coated stainless-steel wires, which were twisted to a tetrode. The active electrode surfaces at the tips were approximately aligned with a distance of 0.5 mm between outer and inner contacts, and 1.0 mm between the two inner contacts. In addition, a single Teflon-coated tungsten wire (60-μm diameter) was used as a reference electrode, whereas the ground electrode was realized by a miniature screw placed in the skull. Prior to implantation, all electrodes were characterized by an impedance analyzer with electrochemical interface (Solartron 1260 and 1287, Farnborough Hampshire, UK) to ensure similar electrical characteristics.

Three more electrodes were implanted in the right hippocampus posterior and anterior to the injection side, and four symmetrical electrodes were implanted into the contralateral hippocampus. All electrodes were placed with their tip in the hippocampus, and only hippocampal structures were covered by the recording. The reference and ground electrodes were placed 1 mm posterior to the lambdoid suture. Electrodes were connected to the head box, and the head box was fixated on the skull using dental cement. Two hours after kainate injection, status epilepticus was interrupted by applying diazepam intraperitoneally (Somerlik et al., 2011).

At the end of the electrophysiological experiments, rats were deeply anesthetized and perfused with 2.5% paraformaldehyde. Brains were removed and placed in 2.5% paraformaldehyde for at least 48 h before histological sectioning and Nissl staining. The correct position of the electrodes was verified by histology after the animals were sacrificed.

### Recording of Video and EEG Data

Video recordings were performed continuously for the duration of the study and screened for seizure activity on a daily basis. An EEG recording was added 72 h after implantation to allow for a postsurgical rest period for the animals. Recording devices were place on the animal’s head in a way that allowed free range of movements. EEG was recorded using MPA81 recording boxes and PGA32 amplifiers (Multi Channel Systems, Reutlingen, Deutschland). The sampling rate was set at 10,000 Hz. Recording of data as well as later analysis for seizures was performed using Spike2 (Cambridge Electronic Design, Cambridge, England). This software was also used for synchronization between video and EEG recordings. Both EEG and video recordings were visually screened for seizure occurrence on a daily basis.

### Behavioral Analysis of Spontaneous Seizures

Rats were included in the current analysis if they showed regular spontaneous seizure after status epilepticus and if they had at least three seizures with mild symptoms (Racine 1–4) and three seizures with severe symptoms (Racine 5&6; Racine, 1972).

Racine scales were used according to Besio et al. (2007) as follows: *R* = 0, no motor seizure activity; *R* = 1, eye closure and masticatory movements; *R* = 2, head nodding; *R* = 3, mild forelimb clonus; *R* = 4, clonus with rearing; *R* = 5, clonus with rearing and falling; and *R* = 6, wild running fit during seizure. Scoring of Racine scales was conducted by two independent reviewers, and seizures were excluded if the reviewers did not agree on scoring.

Seizures were selected according to the following criteria: 4 weeks with regular seizure activity were selected after the time of established regular seizures. In this period, the first seizure of each Racine scale was selected for each week, resulting in a maximum of 24 selected seizures per rat (4 weeks with 6 seizure types maximum). Seizures had to be at least 15 min apart from each other to be selected. All EEG samples of seizures were screened for artifacts prior to applying the high pass filer. As we were only analyzing the two contacts of an intracranial electrode that was lying deep within the hippocampus, artifacts were usually not visible even during severe seizures. Seizures with substantial artifacts have been excluded from the analysis. Figure 1B shows the exemplary selection of seizures for rat 1.

### EEG and HFO Analysis of Seizures

EEG data were converted from the SPIKE2 format to Harmonie Monitoring System (Stellate, Montreal, QC, Canada) for the analysis of the HFO activity. Conversion was performed using the ASA software (ANT Neuro HQ, Enschede, Netherlands), as has been described in previous publications (Kuhnke et al., 2019). Two independent reviewers selected the time of seizure onset and seizure termination for all seizure periods; in cases of disagreement, seizures were discussed together to find a consensus. For each seizure, a 30-s segment directly prior to seizure onset, the whole ictal period, and a 30-s postictal segment were selected for analysis. Seizure onset and termination time were defined using the EEG recording. Seizure onset was set at the first visible ictal activity, which consisted of a high amplitude spike followed by rhythmic spiking activity in our model. Seizure termination was defined as the end of rhythmic activity in conjunction with EEG suppression. EEG analysis was performed using a bipolar montage connecting the two contacts of each electrode. Prior to filtering and HFO analysis, all segments with large and/or continuous artifacts were excluded.

All ictal HFO analysis was performed visually by two independent reviewers. Only events marked by both reviewers were used for the following analysis. In cases in which one of the events was marked differently, only the part of the event identified by both reviewers was taken into account.

For HFO identification, we used previously published criteria and methods (Zijlmans et al., 2017). The display of the computer screen was split vertically to display EEG data high-pass-filtered with 80 Hz (FIR) in the left and high-pass-filtered with 250 Hz on the right. Data were displayed with maximal temporal resolution (0.4 s per page), and the amplitude was increased to 1.5–2 μV/mm. Ripples were marked if an event was visible on the left but not on the right side of the screen. Fast ripples were marked if seen on the right side. Oscillations had to have a minimum of four oscillatory components. Additionally, all ictal spikes as visible on the unfiltered EEG were marked.

### Statistical Analysis of Marked HFO

For each channel, an in-house Matlab (Mathworks, Natick, MA, USA) based program was used to calculate the number of seconds occupied by the HFO per minute for each channel for each of the seizure segments (preictal, ictal, postictal). This resulted in seconds per minute of time covered by the HFO for each segment and each channel. This measure will be referred to as the HFO ratio in the Results section. We selected this measure as seizures were longer with increasing severity, and thus, we felt it was important to use a measure independent of the seizure length.

Different seizure segments were compared between mild seizures (Racine 1–4) and severe seizures (Racine 5&6) in regard seconds per minute of time covered by the HFO. As data were not normally distributed, a Wilcoxon rank sum test was performed for comparison between different seizure severities. The significance level was *p* < 0.05.

## Results

### Occurrence of Spontaneous Seizures Across Animals

In total, 12 rats underwent the described experiments, which were also conducted as part of another research question. No rat died during status epilepticus or during the following observation period; two rats died during the anesthesia process and preparation prior to status epilepticus.

Six of these rats developed regular seizures after 4–8 weeks of observation after status epilepticus. Only two rats (both female) expressed seizures with both severe and mild Racine scores. Rat 1 had 232 nonsevere and 55 severe seizures; rat 9 had 42 nonsevere and 55 severe seizures within the 4-week analyzed period. Overall, time to regular seizures was long with 264 days after status epilepticus in rat 1 and 266 days in rat 2. All seizures observed in the study started over the most interior to contacts of the hippocampal electrode ipsilateral to the kainite injection site.

A total of 151 EEG channels were analyzed during 24 seizures with different Racine states. Three seizures were scored Racine 2 (two from rat 1, one from rat 9), six seizures were scored Racine 3 (three from each rat), and five seizures were scored Racine 4 (three from rat 1, two from rat 9). As all of these seizures met the criteria for focal seizure activity, they were considered mild. Six seizures were scored Racine 5 (three from each rat), and four seizures were scored Racine 6 (two from each rat). Therefore, 10 seizures were categorized as severe.

### Comparison of HFO Occurrence Between Different Seizure Periods

**Figure 2:**
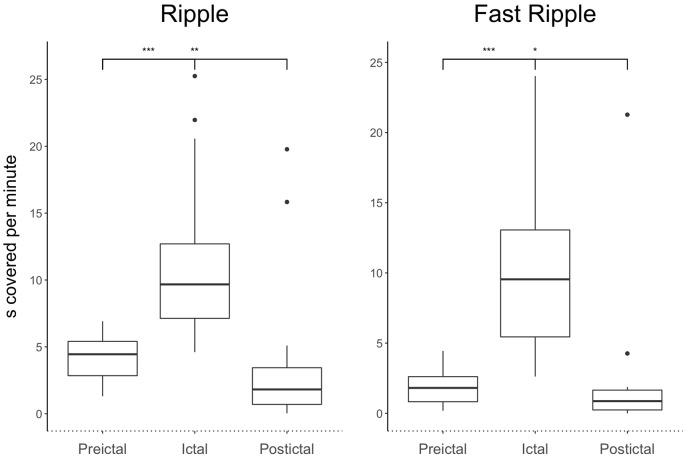
Significant ictal increase in ripples and fast ripples compared to the preictal phase as well as significant postictal decrease across all types of seizure severity. All stars indicate significance *p*, 0.05.

The ratio of ripples and fast ripples was significantly higher during ictal than preictal periods (*p* < 0.0001, *n* = 24). The median duration covered increased by 150% for ictal ripples and by 300% for fast ripples compared to the preictal period. The ratios of ripples and fast ripples significantly decreased in the postictal phase in contrast to the preictal and ictal phases [*p* < 0.03, (*n* = 24)]. The median duration covered decreased by 75% for ripples and 95% for fast ripples in the postictal period compared to the ictal period. A 37% reduction was seen for ripple coverage and 80% for fast ripple coverage when comparing postictal to preictal periods. Figure 2 demonstrates covered time per minute for ripples and fast ripples during the different seizure periods.

### Comparison of HFO Occurrence Between Mild and Severe Seizures

**Figure 3:**
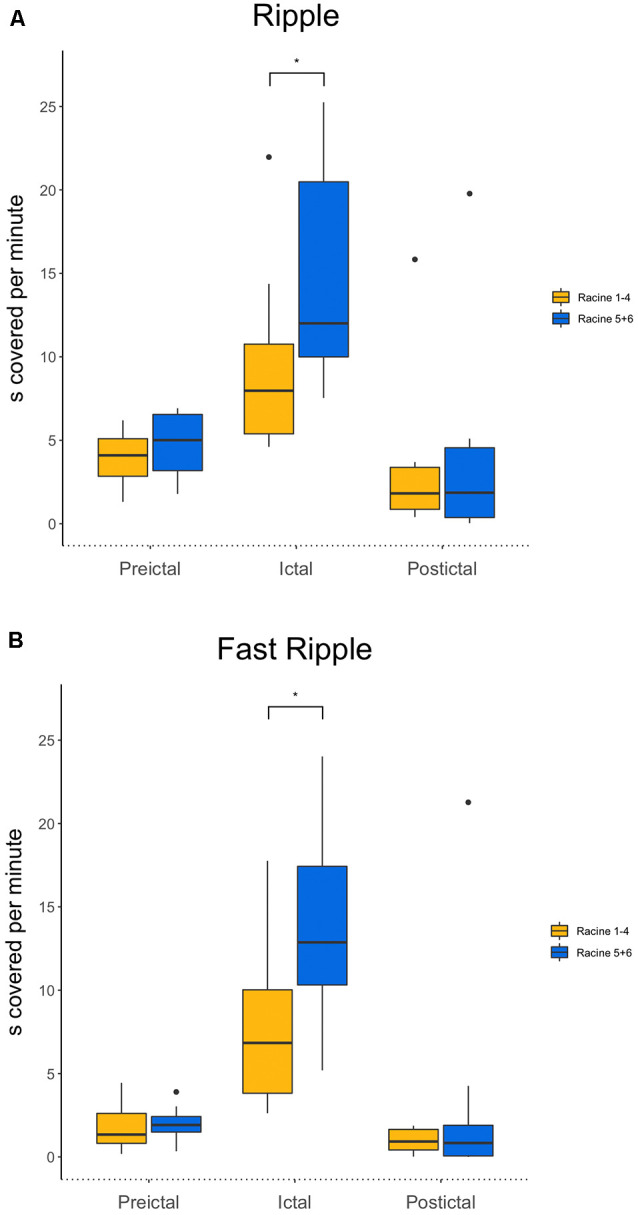
**(A)** Comparison of ripples between mild and strong seizures for all three phases. Ictal ripple occurrence is significantly higher in stronger seizures. **(B)** Comparison of fast ripples between mild and strong seizures for all three phases. Ictal fast ripple occurrence is significantly higher in stronger seizures. *p < 0.05.

Ripples ratios were significantly higher during the seizure when comparing mild with severe seizures (*p* = 0.04, *n* = 10 for severe seizures, *n* = 14 for mild seizures; see Figure 3A). No difference was seen for the preictal and postictal phases.

Fast ripple ratios also were higher during the ictal period during severe compared to mild seizures (*p* = 0.02, *n* = 10 for severe seizures, *n* = 14 for mild seizures). Again, no differences during the preictal and postictal periods were seen for the different seizure strengths (see Figure 3B).

### Analysis of HFO Occurrence in Individual Rats

We repeated the statistical analysis for each rat separately. Rat 1 had 232 nonsevere and 55 severe seizures, and rat 9 had 42 nonsevere and 55 severe seizures within the 4-week analyzed period. The ripple ratio was significantly higher in ictal than preictal periods (rat 1: *p* < 0.001, *N* = 28, Rat 2: *p* < 0.008, *N* = 20). Postictal segments had significantly lower ripple ratios than ictal (Rat 1: *p* < 0.001 *N* = 28, Rat 2: *p* = 0.04, *N* = 20) and preictal (Rat 1: *p* < 0.001, *N* = 28, Rat 2: *p* = 0.02, *N* = 24) periods. The fast ripple ratio was significantly higher in ictal than preictal segments (Rat 1: *p* < 0.001 *N* = 28, Rat 2: *p* = 0.007, *N* = 24) and significantly lower in postictal compared to preictal (Rat 1: *p* < 0.001, *N* = 28, Rat 2: *p* = 0.01, *N* = 24). The ripple ratio was significantly larger in both rats for severe compared to mild seizures (Rat 1: *p* = 0.04 *N* = 42, Rat 2: *p* = 0.03 *N* = 30); no difference was found for fast ripples.

## Discussion

### Result Summary

The present study confirms our hypothesis that HFOs reflect seizure severity and ictal behavior in seizures generated over the hippocampus. HFOs were significantly more frequent during the ictal than the interictal period. After the seizure, a significant suppression of HFOs was seen. In the ictal period, HFOs were significantly higher in seizures with clinical symptoms of generalization than in those that stayed focal with mild symptoms. No difference between different seizure severities in HFO occurrence was seen in the pre- or postictal period.

### Limitations

#### Kainate Model Limitations and Behavioral Analysis of Seizures

In the focal kainate rat model, around 60% to 80% of animals develop spontaneous seizures after a latency period of 15–10 days (Drexel et al., 2012). The majority of these animals should enter a phase of stable chronic epilepsy with regular nonconvulsive seizures around 3 months after initial status epilepticus (Lévesque and Avoli, 2013). All of our animals were observed after status, but only two reached the stadium of chronic epilepsy with regular seizures, which is a limitation of the present study. The success rate of this experiment is largely dependent on the kainate dose as well as the length of status epilepticus. We chose our approach according to previous literature, but success rates might be increased by choosing a high kainate dose in the future (Williams et al., 2009). While we had many EEG channels and seizures to analyze in this analysis, the fact that these all arrived from few animals might reduce the ability to generalize results in the same way as would be possible with more animals that had seizures. We feel, however, that this should not affect the most important information in this study, which is comparing clinically severe to mild seizures, as we analyzed a larger number of seizures.

A rat model can always only approximate epilepsy as it occurs in patients. The kainate model used in this study is considered comparable to human temporal lobe epilepsy, especially if it focuses on a focal hippocampal injection as done here (Lévesque and Avoli, 2013). One could, nevertheless, argue whether analysis from intracranial EEG in patients would be more valuable. Our group has investigated a very similar question in patients with temporal lobe seizures previously (Schönberger et al., 2019). We have, nevertheless, chosen to replicate these findings in a kainite model for two reasons. First, this is a great opportunity to correlate neurophysiological measures like HFOs with behavioral observations in seizures. Second, seizures in rat models of epilepsy can be scored more reliably and in more detail using the Racine scale than in patients in whom seizure semiology is much more variable. Besio had described a sixth grade of severity characterized by wild running in the cage during a seizure. As we observed this phenomenon in our experiment as well, it was decided to apply the modified Racine scale in this study as suggested by Besio et al. (2007). For this study, both the EEG and the video were screened for seizure activity, which allows for maximum sensitivity when it comes to the detection of subtle clinical seizures. Video cameras allowed assessing even small mouth and upper extremity movements. Two independent reviewers were used to ensure that Racine scores were as accurate as possible.

#### Pitfalls of HFO Analysis

HFOs were scored visually during all three time periods around the seizure. It was decided to use visual identification even if this is more time-consuming and bears the danger of reviewer bias as we were looking at ictal HFOs and wanted to exclude the effects of regular ictal spiking on the analysis. While many automatic detection tools exist for interictal HFOs, current automatic detectors are not calibrated for ictal HFOs (Staba et al., 2002; Dümpelmann et al., 2015). Most of the ictal HFO analyses have been working with high-frequency spectra instead of distinct HFOs, and therefore, the methodology did not apply to our analysis (Ochi et al., 2007). We therefore decided to mark ictal spikes and HFOs visually by two independent reviewers and only retained those events identified by both. Ictal HFOs are often longer than interictal HFOs. To account for the large variability in length, we decided to calculate seconds covered by the HFO per minute instead of HFO rates as is usually done (Jacobs et al., 2010; Zijlmans et al., 2011). This is very similar to the methods used in previous studies with human sEEG. Another challenge is the frequency definition of HFOs. Data derived from interictal periods usually distinguish ripples (80–200/250 Hz) from fast ripples (250–500 Hz). While this distinction derived from early microelectrode studies (Staba et al., 2004) and is continuously used for current studies, some studies suggest that the separation is artificial, and many high-frequency events show a strong overlap between these frequencies (Pearce et al., 2013). Moreover, these separations were derived from interictal data, and peri-ictal HFOs might behave differently concerning frequencies. Our results are very similar for ripples and fast ripples, which might suggest that these two event types are not distinct in the way they behave during seizures.

The influence of the electrode contact size on the recording of HFOs has been debated controversially, and as in most animal models, the electrodes used for the recording in this study have a smaller contact size than the electrodes used in humans. Contact size is especially critical when using HFO recordings from different electrodes within the same study as this might bias the results. In our study, all electrodes had the same size, and the results should be comparable. Moreover, two studies on the effects of the contact size of HFO rates showed no difference between different contact sizes and HFO identification, one using human data and one in an epilepsy model (Châtillon et al., 2011, 2013).

Analysis of HFOs always struggles with the fact that both physiological and epileptic oscillations within the same frequency range can occur in humans as well as rats. Interictal- and task-related physiological HFOs have been described during eye and mouth movements in patients (Uematsu et al., 2013; Nakai et al., 2017), and occurrence of these physiological oscillations is hard to test in an animal model. We feel, however, relatively confident that the majority of events identified in the present study were epileptic as they mainly occurred within the SOZ, and our study focused on ictal HFOs, which are less likely to be physiological.

### Pathophysiological Importance of HFO Mirroring Seizure Severity

#### Correlation Between HFO Occurrence and Different Seizure Periods

This study shows similar results in the hippocampus for rodents with chronic epilepsy as have been described in patients with refractory focal epilepsy. Several studies indicate that HFOs increase during the ictal period compared to the interictal period. In our study, no difference was found for ripples vs. fast ripples. This is similar to the data from Zijlmans et al. (2011) that showed a significant increase in ictal HFOs compared to the preictal and interictal phases. In the current study, we did not compare ictal and preictal HFOs to an interictal EEG segment. Overall data about the preictal HFO increase are less conclusive than those about the ictal HFO increase. An increase in HFOs prior to the occurrence of seizure would, of course, be clinically highly relevant as it could be used for seizure detection and warning, but most preictal HFO increases described in the literature occurred in the very few seconds directly prior to the seizure onset and therefore were clinically less relevant. This study also analyses the immediate postictal period. Similar to the data from patients with mesio-temporal epilepsy, a significant decrease in HFOs overall was seen in the postictal period. This most likely reflects a postictal suppression of epileptic activity. Similar observations have been found for interictal HFOs in the postspike slow wave (Urrestarazu et al., 2007).

#### Correlation Between HFO Occurrence and Seizure Severity

Data from patients with mesio-temporal epilepsy also suggested a more widespread and higher increase in HFOs in patients who had secondary generalized compared to focal seizures (Schönberger et al., 2019). In rats, seizures were shorter and showed faster evolution from focal to generalized, which is why a separation of two ictal periods was not possible. However, ictal HFOs were clearly higher in the ictal period during severe clinical seizures than milder ones. Racine states considered severe were those whose clinical appearance equaled generalized seizures in humans. Therefore, the observed difference for HFOs for generalized vs. focal seizures during the ictal periods was similar in both rats and patients. Overall, these observations suggest that HFOs mirror the epileptic activity during active seizures. Moreover, they are an EEG correlate of seizure severity.

One could hypothesize that stronger or generalized seizures lead to a more substantial suppression of HFOs in the postictal period; however, this could neither be found for patients not in our animal model. So far, no systematic studies have investigated the length and degree of postictal HFO suppression, and this might be a future topic of discussion to better understand postictal recovery. It is important to note that the current study only focuses on the hippocampal region, which was covered during the experiments; we cannot, therefore, draw any conclusion on neocortical HFOs and seizure severity.

## Conclusion

The present study investigated the relationship between seizure severity as determined by clinical semiology and HFO occurrence. It could be clearly shown that HFOs correlate with increasing seizure severity. This can be seen as evidence that HFOs mirror seizure severity and provides a link between the behavioral seizure observation and HFOs as a measurable EEG biomarker.

## Data Availability Statement

The raw data supporting the conclusions of this article will be made available by the authors, without undue reservation.

## Ethics Statement

The animal study was reviewed and approved by Institutional Animal Care and Use Committee of the University of Freiburg.

## Author Contributions

NB and JS performed EEG and statistical analysis. KS-F performed animal experiments under support and supervision of AS-B. JJ supported EEG analysis, was responsible for concept and hypothesis in study and supported NB in writing the manuscript. All authors contributed to the article and approved the submitted version.

## Conflict of Interest

The authors declare that the research was conducted in the absence of any commercial or financial relationships that could be construed as a potential conflict of interest.
